# Perceived Applicability of Value-Based Healthcare in Military Health Systems: Results From a Pilot Survey Study

**DOI:** 10.1177/00469580261427434

**Published:** 2026-03-08

**Authors:** Henk van der Wal, Veronika Sedivcova, Fleur Maas, Brett Nishikawa, Diane Lamb, Iris Dijksma, Jacopo Frassini, Marián Ivan, Rigo Hoencamp, Hynek Schvach

**Affiliations:** 1 University Medical Center Rotterdam, The Netherlands; 2 Ministry of Defence, Utrecht, The Netherlands; 3 University of Defence, Hradec Kralove, The Czech Republic; 4 United States Air Force School of Aerospace Medicine, Wright-Patterson Air Force Base, Dayton, OH, USA; 5 Royal Centre for Defence Medicine, Birmingham, UK; 6 Aeronautica Militare, Roma, Lazio, Italy; 7 NATO Centre of Excellence for Military Medicine, Budapest, Hungary

**Keywords:** value-based healthcare, military health services, healthcare delivery, Europe, eastern, perception, pilot projects

## Abstract

Value-Based Healthcare (VBHC) is gaining traction in civilian systems, but its relevance and feasibility for Military Health Systems (MHSs) in Central and Eastern Europe (CEE) remain unclear. This pilot study explored familiarity, perceived applicability and desirability of VBHC among military healthcare stakeholders. A pilot cross-sectional perception study was conducted during the 2024 VIMIMED Military Medicine Conference, combining a brief expert introduction with a structured survey. The survey assessed baseline familiarity, perceived applicability in home-base and operational care, and desirability of VBHC implementation. Descriptive statistics were used. The association between familiarity and desirability was explored using Fisher’s exact test. Among 65 workshop participants, 37 completed the survey. Over half of respondents reported low baseline familiarity with VBHC (51.4%). Despite this, VBHC was widely perceived as desirable (89.1%). No statistically significant association was found between familiarity and desirability (Fisher’s exact test, *P* = .672). Thirty-five respondents considered VBHC applicable in at least one domain and were included in component-level analyses. The components “multidisciplinary team,” “educate, innovate & improve,” and “IT & data” were most frequently endorsed as applicable. Respondents who perceived VBHC as applicable in both home-base and operational care tended to endorse more components than those who perceived applicability in home-base care only. Despite limited baseline familiarity, VBHC was widely perceived as desirable and contextually applicable within CEE MHSs. These exploratory findings suggest potential for targeted, phased integration of selected VBHC components. Larger and, more representative studies are needed to assess implementation feasibility, pathways, and sustainability of VBHC in MHSs.

## Introduction

Value-Based Healthcare (VBHC), introduced by Porter and Teisberg in 2006, focuses on delivering high-value care measured by patient outcomes rather than the volume of services delivered.^1,2^ The transformation to VBHC has already taking place on a broader scale in countries like the United States, United Kingdom, and the Netherlands,^3,4^ implementing overarching change strategies. The transformation towards high-value care has been guided by Porter’s “Value Agenda,”^5^ a strategic framework consisting of interdependent and mutually reinforcing components that achieve the greatest progress when implemented together. In the Netherlands, the Linnean Initiative,^6^ expanded these to 7 components, shifting towards elements as leadership and culture, but also education, and innovation. Important additions to strengthen further operationalisation of VBHC are “patient-centred care” and “shared decision-making.”^7,8^ Supplemental Material S1 provides an overview of both Porter’s Value agenda and Linnean VBHC components.

Contrasting VBHC with military healthcare reveals both overlap and divergence. VBHC emphasises patient-centred outcomes, transparency and value creation, whereas military healthcare traditionally focuses on collective readiness and mission success. Nevertheless, both aim to optimise outcomes making use of available resources. Other studies have shown that several VBHC components—such as multidisciplinary teamwork, outcome measurement and continuous improvement—could enhance military healthcare performance, bridging individual recovery with organisational readiness.^9^ In 2023, the military relevance of VBHC has been highlighted through an in-depth exploratory analysis by the NATO Science and Technology Organisation (STO),^10,11^ which identified its potential to improve healthcare quality, return-to-duty rates and resource efficiency while strengthening civil-military learning and collaboration.^12-14^ Despite these promising insights, VBHC implementation within MHSs remains limited and fragmented.

This pilot study aimed to assess the perceived feasibility of VBHC within the MHSs of 4 Central and Eastern European (CEE) countries—the Czech Republic, Hungary, Poland and Slovakia—by exploring the familiarity, applicability and desirability of VBHC among military healthcare professionals and leaders. The following research question was addressed: “How do military healthcare providers and leaders in the 4 CEE countries perceive the familiarity, applicability, and desirability of VBHC?”

## Methods

### Study Context and Design

**Figure 1.**
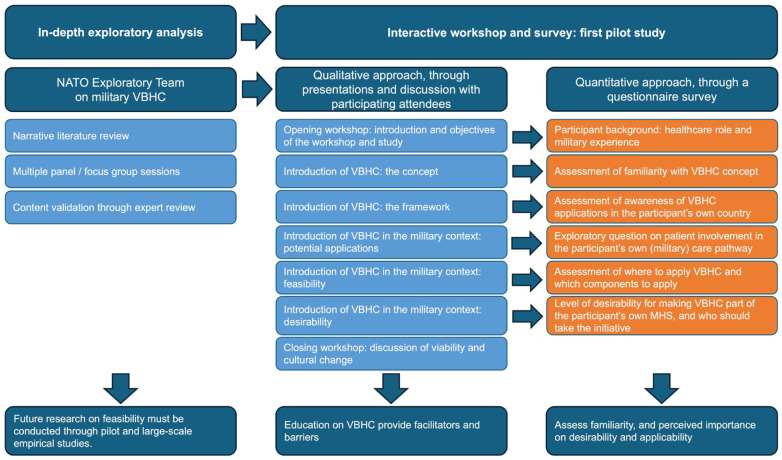
Overview of the study design and data collection procedure. The study followed a sequential design consisting of an introductory expert session, a structured perception survey, and a facilitated group discussion conducted during an interactive workshop. Participants first reported baseline familiarity with value-based healthcare (VBHC), followed by a brief introduction to VBHC in a military context. Subsequently, perceived applicability of VBHC and its components was assessed, and finally participants rated the desirability of implementing VBHC within their own military health system.

The study design is illustrated in Figure 1, which outlines the sequential approach comprising an introductory expert session, a structured perception survey, and a facilitated group discussion. The design integrated a cross-sectional survey component with an interactive workshop, enabling both quantitative assessment and qualitative reflection on the perceived familiarity, applicability, and desirability of VBHC in military context. This design was selected to obtain a first empirical overview of how MHS stakeholders—from healthcare providers to leadership personnel—understand and evaluate the potential relevance of VBHC within their operational and organisational environments, thereby informing future feasibility and implementation studies. The reporting follows the Strengthening the Reporting of Observational Studies in Epidemiology (STROBE) guidelines.^15^

### Setting and Participants

The study commenced with the introductory expert session conducted by the exploratory team established by NATO STO, initiated to analyse the feasibility and military relevance of VBHC across NATO MHSs. The multinational team consisted of participants from the Netherlands, the United Kingdom, the United States, the Czech Republic, Italy, and a representative from the NATO Centre of Excellence for Military Medicine. Building upon that multinational foundation, the cross-sectional survey was carried out during an interactive workshop at the 2024 Visegrad Military Medicine (VIMIMED) Conference in Prague. The conference, co-organised by the military medical services of the Czech Republic, Hungary, Poland, and Slovakia, provided a unique forum for regional cooperation and exchange on military health topics, making it an appropriate venue to explore perceptions of VBHC.

Eligible participants included active-duty service members involved in healthcare delivery, leadership, planning, or health system management within their respective MHSs. Convenience sampling was applied, drawing on attendees who voluntarily joined the VBHC workshop and completed the survey. Participants reflected the multidisciplinary composition of MHSs, including clinicians, administrators, medical commanders, and potential care recipients, making it able to obtain rapid, situational insights. This sampling strategy does not yield a representative population sample, but it was considered suitable for the exploratory and feasibility-oriented nature of this pilot study. A pragmatic participation target of at least 35% of attendees was defined a priori, with the aim of ensuring a sufficiently broad representation of perspectives within the workshop audience.^16^ Prior to participation, individuals were informed about the study aims and procedures and written informed consent was obtained. All data were collected anonymously to encourage open reflection on familiarity with, and attitudes towards, VBHC in both home-base and operational military contexts.

### Survey Method & Exploratory Workshop

Participants were asked to complete a questionnaire from the perspective of their own professional background and military experience. Perception was operationalised as participants’ subjective evaluation of the extent to which VBHC components, adapted from the Linnean Initiative domain descriptions,^6,17,18^ could be applied within both home-base (non-operational) and operational (deployed) care settings. The questionnaire was developed by 2 of the researchers (HW; FM) with recommendations from the introductory expert session, and earlier findings from van der Wal et al,^9^ with human-centred insight exploration elements of the Design Thinking framework by Brown and Kātz.^19^ The questionnaire was developed based on 2 underlying principles: a perception survey,^20^ which focuses on subjective opinions and perceived advantages and disadvantages; and a stakeholder assessment survey,^21^ which gathers input from a variety of perspectives based on participants’ roles. All participants were provided with identical instructions and were asked to complete the questionnaire simultaneously following the brief, neutral introduction to VBHC, with the aim of reducing differential exposure and ensuring standardised administration. The nature of the workshop format, which was designed to accommodate a single time point and a restricted window for data collection, necessitated the instrument to be both concise and exploratory in design. As shown in Figure 1, the workshop was designed with participants being asked about baseline familiarity with VBHC, followed by an introduction to VBHC in a military context. The remaining questions were asked following the initial introduction of VBHC. Finally, at the end of the workshop, participants were asked to rate the perceived desirability of VBHC.

Familiarity with VBHC and the desirability of its implementation were assessed using 10-point numeric rating scales ranging from 1 (not at all) to 10 (completely). Only the anchor points of the scale were labelled, with intermediate values deliberately left unlabelled to give respondents the freedom to position their judgement along a continuous subjective spectrum. No explicit neutral midpoint was provided. This was a deliberate approach, as the study aimed to elicit directional judgements following a brief, standardised introduction to the concept of VBHC rather than measuring established or stable attitudes. Component-level applicability was assessed using binary response options (yes/no).

### Data Collection Procedure and Recruitment

We surveyed conference participants in-person using a combination of Microsoft PowerPoint and an audience interaction platform, *Slido* (see Supplemental Material S2 for the questionnaire and Supplemental Material S3 for the workshop outline and topics & survey questions). Respondents were presented an informed consent and a brief introduction to the aim of the survey before completing the questionnaire. The surveys were anonymous, participation was voluntary, and all data was handled by one author (HW). There were no exclusion criteria for participation.

### Analysis

Survey data were exported from the *Slido* platform (Slido for Mac v1.1) and analysed using *IBM SPSS Statistics version 29.0* (IBM Corp., version 29.0.0.0 (241), Armonk, NY, USA). Descriptive statistics summarised participant characteristics and distributions of responses related to familiarity with VBHC, perceived applicability, and desirability of implementation. Responses for the 10-point scales (familiarity and desirability) were analysed using the full 1 to 10 range without grouping. In addressing the domain-of-applicability question, respondents were invited to indicate their perception of VBHC’s applicability in the following domains: (1) home-base care, (2) operational care, (3) both, or (4) neither. Accordingly, the participants were categorised into 4 mutually exclusive groups, namely: home-base only, operational only, both domains, or neither. The calculation of percentages was based on these mutually exclusive categories, without aggregation across groups.

Component-level applicability was assessed using binary (yes/no) items, indicating whether respondents considered each component applicable. Respondents who indicated that VBHC was “not applicable in any domain” (n = 2) were excluded from subsequent component-level analyses, as their responses could not meaningfully contribute to the assessment of perceived applicability of individual VBHC components. Consequently, component-level analyses were conducted on the remaining sample (n = 35). To examine the association between familiarity with VBHC and the desirability of its implementation, both variables were categorised for inferential analysis. Initial inspection of contingency tables based on the full-scale response categories revealed sparse tables with low expected cell counts. Therefore, for inferential purposes, both variables were dichotomised into “low” and “high” categories and analysed using a 2 × 2 contingency table. Given the remaining small cell counts, Fisher’s exact test was used instead of the Pearson chi-square test.

## Results

A total of 65 participants actively participated in the interactive workshop, of whom 37 completed the survey in full, and were included in the primary analyses. The respondent group consisted of military healthcare providers (n = 18), military healthcare leadership and support personnel (n = 17), and other participants or potential care recipients (n = 2). Demographic characteristics of the participants are presented in Table 1.

**Table 1.** Demographics of the Survey Participants at the VIMIMED VBHC Workshop.

Role in military healthcare				
Other / military patient (potential care recipient)	Military healthcare professional (healthcare provider)		Military leadership (healthcare support)	
5.4% (n = 2)	48.6% (n = 18)		45.9% (n = 17)	
Years of experience within defence				
0-15 years		≥15 years		
37.8% (n = 14)		62.2% (n = 23)		
Country represented				
Czech Republic	Slovakia	Hungary	Poland	Other
51.4% (n = 19)	13.5% (n = 5)	16.2% (n = 6)	16.2% (n = 6)	2.7% (n = 1)

### Familiarity With VBHC

Participants rated their prior familiarity with VBHC on a numeric scale ranging from 1 (not at all familiar) to 10 (completely familiar). A total of 37 respondents provided a valid response to this item. The distribution of responses across the full scale was as follows: score 1, n = 12 (32.4%); score 2, n = 5 (13.5%); score 3, n = 2 (5.4%); score 4, n = 2 (5.4%); score 5, n = 3 (8.1%); score 6, n = 1 (2.7%); score 7, n = 2 (5.4%); score 8, n = 6 (16.2%); score 9, n = 1 (2.7%); and score 10, n = 3 (8.1%). Interpreted descriptively, 51.4% (n = 19) rated their familiarity in the lowest range (scores 1-3), corresponding to being not at all to slightly familiar with VBHC. A smaller proportion placed themselves in the middle of the scale (scores 4-6), corresponding to somewhat to moderately familiar (16.2%, n = 6), while approximately one-third reported higher familiarity (scores 7-10), corresponding to very to completely familiar (32.4%, n = 12). When asked whether they were aware of any existing implementation of VBHC in their own country’s civilian or military healthcare system, 17 respondents (45.9%) answered affirmatively. In addition, 29 respondents (78.4%) agreed that the patient should be involved in their own care pathway.

### Desirability of VBHC Implementation in Military Healthcare

Respondents rated the desirability of making VBHC part of their own military health system on the same numeric scale ranging from 1 (not at all desirable) to 10 (completely desirable). All 37 respondents provided a valid response to this item. The distribution of responses across the full scale was as follows: score 1, n = 1 (2.7%); score 2, n = 1 (2.7%); score 3, n = 2 (5.4%); score 4, n = 2 (5.4%); score 5, n = 10 (27.0%); score 6, n = 4 (10.8%); score 7, n = 6 (16.2%); score 8, n = 8 (21.6%); score 9, n = 1 (2.7%); and score 10, n = 2 (5.4%). To examine the association between familiarity with VBHC and the desirability of its implementation, both variables were dichotomised into low and high categories and analysed using Fisher’s exact test due to small cell counts. No statistically significant association was observed between the 2 variables (Fisher’s exact test, 2-sided *P* = .672; n = 30).

### Perceived Applicability of VBHC Within Military Health Systems

**Figure 2.**
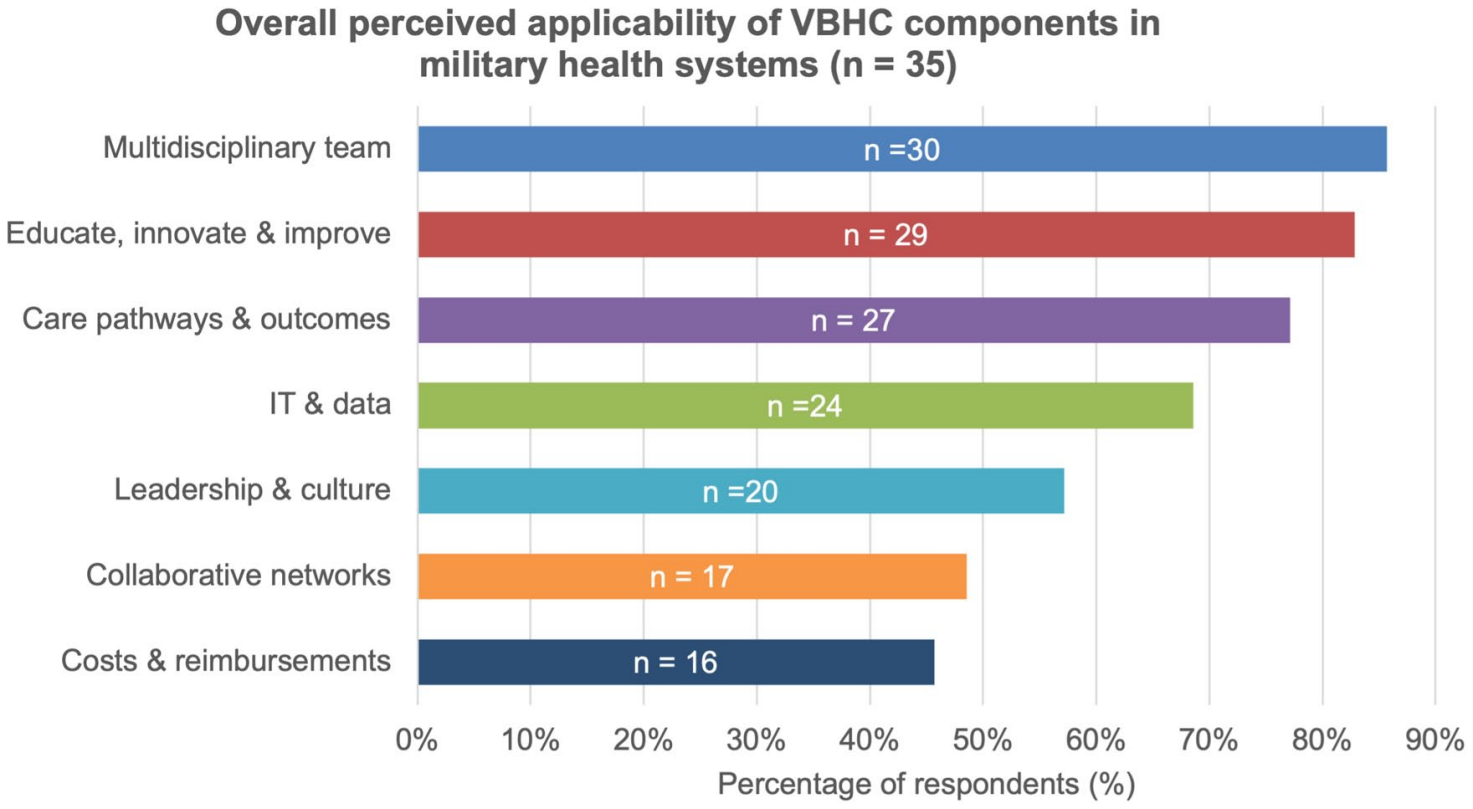
Overall perceived applicability of VBHC components within military health systems. Bars represent the percentage of respondents indicating each component as applicable (yes/no). Absolute numbers of respondents (n) are shown within the bars. Two respondents who indicated that VBHC was not applicable in any domain were excluded from this analysis (n = 35).

**Figure 3.**
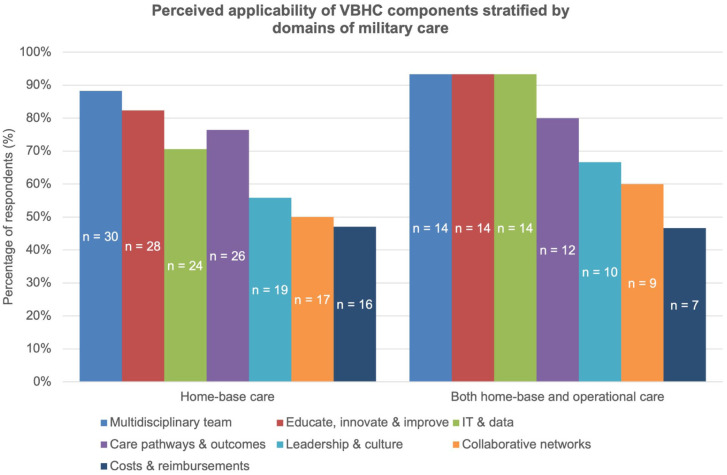
Perceived applicability of VBHC components stratified by domain of military care. Bars represent the percentage of respondents indicating each component as applicable (yes/no), shown separately for respondents who perceived VBHC as applicable in home-base care only and for those who perceived VBHC as applicable in both home-base and operational care. Absolute numbers of respondents (n) are shown within the bars. Two respondents who indicated that VBHC was not applicable in any domain were excluded from this analysis (n = 35).

Participants were first invited to indicate in which domain(s) they perceived VBHC to be applicable: home-base care, operational care, both (home-base and operational care), or neither. Based on mutually exclusive categorisation, 19 respondents (51.4%) indicated applicability in home-base care only, 2 respondents (5.4%) in operational care only, 14 respondents (37.8%) in both domains, and 2 respondents (5.4%) indicated that they did not perceive VBHC to be applicable in either domain. Respondents who indicated that VBHC was not applicable in any domain (n = 2) were excluded from further applicability analyses. Consequently, all subsequent results are based on the remaining 35 respondents. These respondents were then asked to evaluate the perceived applicability of each VBHC component using a binary (yes/no) response format. The number of components endorsed as applicable per respondent is presented in the Supplemental Material S4. As illustrated in Figure 2, the overall proportion of respondents indicating the perceived applicability of each VBHC component within their military health system is demonstrated. As presented in Figure 3, the component-level results are stratified by domain-of-applicability group, categorised as home-base care only versus both home-base and operational care.

### Initiative to Implement VBHC

When asked who should own the initiative to implement VBHC in their own MHS, whether the direct care team (bottom-up approach), or MHS leadership (top-down approach), 28 respondents (75.7%) indicated that this should be a shared responsibility between care teams and leadership. Five respondents (13.5%) indicated a preference for a primarily bottom-up approach, and 4 respondents (10.8%) indicated a preference for a top-down approach.

## Discussion

This study assessed the feasibility of VBHC within MHSs amongst military healthcare providers and leadership in a selected group of CEE countries. Identifying the perceived desirability and potential practical implications of (components of) VBHC. The findings suggest that while initial familiarity with VBHC was generally low, even brief exposure to the concept was associated with a strong perceived interest in applying its principles within one’s own MHS. The findings of this study are consistent with those of previous research in civilian settings, where nurses and nursing managers have described VBHC concepts as meaningful but often abstract, requiring contextual interpretation before they can be applied in practice.^22^ Following the introduction to the concepts of VBHC, participants generally considered VBHC to be desirable and applicable in both home-base and operational care settings, particularly regarding the components of: (1) multidisciplinary team, (2) educate, innovate and improve, and (3) IT & data. The high endorsement of these components suggests an endorsement towards integrated, learning-oriented, and multidisciplinary approaches to care delivery. The comparatively reduced number of cost-related components may reflect the need for increased policy-level support, organisational incentives, or capacity-building in future VBHC initiatives. These patterns suggest that a targeted, phased approach involving further research and pilot studies could help to effectively integrate (parts of) VBHC into MHSs. The readiness of armed forces is also based on the education and training of military personnel, a principle reflected in the survey’s high ratings for the VBHC component entitled “Educate, Innovate & Improve.” From this basis, the principles of VBHC may be meaningful embedded within existing military training and education to increase familiarity and shared understanding.

The implementation and feasibility research that is commonly undertaken utilises structured frameworks for the purpose of interpreting early-stage interventions and complex system change. In this context, the Medical Research Council (MRC) Process Evaluation Framework provides a conceptual structure for understanding how interventions are introduced, how they interact with context, and how mechanisms of impact and implementation processes shape outcomes. The Consolidated Framework for Implementation Research (CFIR) provides a comprehensive classification system of contextual determinants that influence implementation success, including organisational, professional, and system-level factors. The RE-AIM framework (Reach, Effectiveness, Adoption, Implementation, and Maintenance) complements these perspectives by focussing on population-level impact and sustainability. Even though the present study was not conceptualised as a formal implementation study, these frameworks offer useful lenses for interpreting the observed patterns of familiarity, perceived applicability, and desirability as early indicators of contextual readiness and potential adoption pathways for VBHC within MHSs.

The exploratory design of this pilot study aligns most closely with the MRC Process Evaluation perspective, which emphasises understanding early responses to interventions and the contextual factors shaping feasibility. The observed patterns in familiarity, perceived applicability, and desirability also reflect CFIR)-relevant contextual determinants, with partial alignment to RE-AIM domains such as reach and early adoption. Evidence from civilian systems shows that differing perceptions of “value” influence professionals’ willingness to engage with VBHC.^23^

NATO’s Continuum of Care model highlights civil-military health cooperation as a means to enhance military health capacity amidst geopolitical developments. The study focuses on the following 2 conclusions from the military relevance analysis conducted by the exploratory team^11^: first, “Better health outcomes and better quality of life for the service members after battle injury (BI) or disease and non-battle injury (DNBI),”^24^ and second, “Effective use of military medical resources and procedures, to maintain the people within the fighting force and minimising money spent on healthcare.”^25,26^ The exploratory team considered these elements to have particular benefit using VBHC to test and advance the effectiveness of MHSs. While VBHC is becoming more widely known in Western countries’ civilian health systems, its implementation is still emerging.^27,28^ Given these findings, we have concluded that the combination of using an expert panel, and a pilot-study consisting of an exploratory workshop and a survey can be an effective way to increase awareness of VBHC within the military healthcare community and to increase the use of VBHC, or some of its component parts.

To further understand the potential role of VBHC in an MHS, it is helpful to consider the uniqueness of the military healthcare context. Unlike civilian healthcare, an MHS places the greatest emphasis on providing care for the largest number of patients to maximise the return-to-duty rate, and operational readiness. In the operational setting, a MHS focuses on “doing the most for the most,” whereas home-base care typically allows greater focus on individualised outcomes. Despite endorsing the view of “doing the most for the most,” there was strong support (78.4%) for involving the service members in their own care pathway: patient-centred care. Although support emerges for patient-centred care, follow-up research needs to enable a better understanding of the feasibility of patient-centred care in an operational context where return-to-duty is likely to be an important factor. Patient engagement can also help patients to improve their care pathway, by integrating them into the MHS so as to co-create suitable learning environments for the (military) caregivers and healthcare leadership.^29^

### Limitations

Pilot studies are primarily designed to assess the feasibility of methods and procedures for larger research.^30^ The main limitation of the study was the modest sample size and the uneven distribution of participants across groups, reflecting voluntary participation during a single professional workshop. This small sample size also necessitated the collapsing of variables for inferential analysis, limiting the granularity of statistical testing. As a result, the findings should be interpreted cautiously and primarily as exploratory.

Therefore, the data are subject to selection bias, given that attendees with an interest in innovation or strategic development may have been more motivated to participate. It is also possible that exposure bias occurred due to the fact that all participants received a brief introduction to VBHC immediately prior to completing the survey, which may have introduced bias and affected their perceptions. The use of 10-point scale without a neutral midpoint may have encouraged respondents to express a directional judgement. While this is appropriate for exploratory feasibility work, this approach may limit comparability with studies using balanced Likert scales. These factors limit the external validity and preclude generalisation to all personnel within the participating MHSs. Furthermore, it should be noted that the questionnaire was newly developed for the present study and had not undergone formal validation. Despite the content validity being reinforced through the process of expert review, the absence of psychometric testing necessitates the interpretation of results as exploratory perceptions rather than stable or fully established attitudes. The time constraints inherent in the workshop context imposed limitations on the depth of questioning and the capacity for repeated measurement. Convenience sampling and the conference setting may have introduced contextual influences not present in everyday clinical or organisational environments. It is evident that these limitations serve to underscore the study’s primary focus on feasibility, thereby highlighting the necessity for future research to employ validated instruments, adopt more rigorous sampling approaches, and encompass a more extensive population coverage, including the patient and partner/family.

A survey of 22 EU countries (amongst Hungary and Poland) showed that only a limited number of countries, such as the early adopters Germany, Sweden, the Netherlands, and the United Kingdom (as former EU country), are leading in VBHC.^31^ Given the potentially limited awareness and barriers and constraints affecting the implementation of VBHC in various CEE countries,^32,33^ there may also be a limitation in the interpretation of the concept of VBHC and possible opportunities for implementation in an MHS. Research on VBHC requires also the inclusion of a relevant sample of patients, including partner and/or family. In this study, the emphasis was placed on participation from the functional role of the participants, as opposed to the perspective of a potential patient. This is even though every military personnel in their MHS is also a potential patient. We also have to take into account that some of the participants, especially junior level, are likely unaware of system-level considerations in their MHS that would be relevant to VBHC feasibility.

## Conclusion

This pilot study offers preliminary insight into awareness and perceived feasibility of VBHC within MHSs in 4 CEE countries. Although baseline familiarity was limited, respondents identified several VBHC components as both desirable and practically applicable within their organisational context. While exploratory and based on a small, non-representative sample, the findings suggest that VBHC principles align with existing priorities in military healthcare. The results also point to an aspirational potential for VBHC to strengthen outcome-oriented and patient-centred practices, provided implementation is carefully adapted to operational constraints. Future research should involve larger, and more balanced samples, include policy-level stakeholders and patient perspectives, and apply longitudinal or mixed-method designs to assess evolving familiarity and feasibility, and to determine whether VBHC can be sustainably integrated into MHSs.

## Supplemental Material

sj-pdf-1-inq-10.1177_00469580261427434 – Supplemental material for Perceived Applicability of Value-Based Healthcare in Military Health Systems: Results From a Pilot Survey Study Supplemental material, sj-pdf-1-inq-10.1177_00469580261427434 for Perceived Applicability of Value-Based Healthcare in Military Health Systems: Results From a Pilot Survey Study by Henk van der Wal, Veronika Sedivcova, Fleur Maas, Brett Nishikawa, Diane Lamb, Iris Dijksma, Jacopo Frassini, Marián Ivan, Rigo Hoencamp and Hynek Schvach in INQUIRY: The Journal of Health Care Organization, Provision, and Financing

sj-pdf-2-inq-10.1177_00469580261427434 – Supplemental material for Perceived Applicability of Value-Based Healthcare in Military Health Systems: Results From a Pilot Survey Study Supplemental material, sj-pdf-2-inq-10.1177_00469580261427434 for Perceived Applicability of Value-Based Healthcare in Military Health Systems: Results From a Pilot Survey Study by Henk van der Wal, Veronika Sedivcova, Fleur Maas, Brett Nishikawa, Diane Lamb, Iris Dijksma, Jacopo Frassini, Marián Ivan, Rigo Hoencamp and Hynek Schvach in INQUIRY: The Journal of Health Care Organization, Provision, and Financing

sj-pdf-3-inq-10.1177_00469580261427434 – Supplemental material for Perceived Applicability of Value-Based Healthcare in Military Health Systems: Results From a Pilot Survey Study Supplemental material, sj-pdf-3-inq-10.1177_00469580261427434 for Perceived Applicability of Value-Based Healthcare in Military Health Systems: Results From a Pilot Survey Study by Henk van der Wal, Veronika Sedivcova, Fleur Maas, Brett Nishikawa, Diane Lamb, Iris Dijksma, Jacopo Frassini, Marián Ivan, Rigo Hoencamp and Hynek Schvach in INQUIRY: The Journal of Health Care Organization, Provision, and Financing

sj-pdf-4-inq-10.1177_00469580261427434 – Supplemental material for Perceived Applicability of Value-Based Healthcare in Military Health Systems: Results From a Pilot Survey Study Supplemental material, sj-pdf-4-inq-10.1177_00469580261427434 for Perceived Applicability of Value-Based Healthcare in Military Health Systems: Results From a Pilot Survey Study by Henk van der Wal, Veronika Sedivcova, Fleur Maas, Brett Nishikawa, Diane Lamb, Iris Dijksma, Jacopo Frassini, Marián Ivan, Rigo Hoencamp and Hynek Schvach in INQUIRY: The Journal of Health Care Organization, Provision, and Financing

sj-pdf-5-inq-10.1177_00469580261427434 – Supplemental material for Perceived Applicability of Value-Based Healthcare in Military Health Systems: Results From a Pilot Survey Study Supplemental material, sj-pdf-5-inq-10.1177_00469580261427434 for Perceived Applicability of Value-Based Healthcare in Military Health Systems: Results From a Pilot Survey Study by Henk van der Wal, Veronika Sedivcova, Fleur Maas, Brett Nishikawa, Diane Lamb, Iris Dijksma, Jacopo Frassini, Marián Ivan, Rigo Hoencamp and Hynek Schvach in INQUIRY: The Journal of Health Care Organization, Provision, and Financing

## References

[1] Porter ME. What is value in health care? N Engl J Med. 2010;363(26):2477-2481. 10.1056/NEJMp1011024 21142528

[2] Porter ME Teisberg EO. Redefining Health Care: Creating Value-Based Competition on Results. Harvard Business Press; 2006.

[3] van Staalduinen DJ van den Bekerom P Groeneveld S Kidanemariam M Stiggelbout AM van den Akker-van Marle ME. The implementation of value-based healthcare: a scoping review. BMC Health Serv Res. 2022;22(1):270. 10.1186/s12913-022-07489-2 35227279 PMC8886826

[4] Cossio-Gil Y Omara M Watson C , et al. The roadmap for implementing value-based healthcare in European university hospitals - consensus report and recommendations. Value Health. 2022;25(7):1148-1156. 10.1016/j.jval.2021.11.1355 35779941

[5] Porter ME Lee TH. The strategy that will fix health care. Harv Bus Rev. 2013;91(10):1-19.

[6] Hanselaar T, van der Kemp M, Wiersma V, van der Linde M, Bos WJ. Getting started with value-based healthcare. Linnean Initiative. 2022.

[7] Tonelli MR Sullivan MD. Person-centred shared decision making. J Eval Clin Pract. 2019;25(6):1057-1062. 10.1111/jep.13260 31407417

[8] van der Nat PB . The new strategic agenda for value transformation. Health Serv Manage Res. 2022;35(3):189-193. 10.1177/09514848211011739 33900128 PMC9277321

[9] van der Wal H Duijnkerke D Engel MFM Hoencamp R Hazelzet JA. Value-based healthcare from a military health system perspective: a systematic review. BMJ Open. 2024;14(11):e085880. 10.1136/bmjopen-2024-085880 PMC11605841 39613433

[10] About the NATO science & technology organization. 2024. https://www.sto.nato.int/Pages/organization.aspx

[11] NATO-STO-HFM-Panel. Technical activity proposal ‘The Applicability of the Value-Based Healthcare Concept’ in NATO (operational) military health systems (HFM-384 ). NATO STO. 2024. https://www.sto.nato.int/_layouts/listform.aspx?PageType=4&ListId={B2AAC100-BE82-43CE-886F-0A467BD6BAA9}&ID=17608

[12] Dombrádi V Bíró K Jonitz G Gray M Jani A. Broadening the concept of patient safety culture through value-based healthcare. J Health Organ Manag. 2021;35:541-549. 10.1108/JHOM-07-2020-0287 33645172

[bibr13-00469580261427434] Kellermann AL Kotwal RS Rasmussen TE. Military medicine’s value to US health care and public health: bringing battlefield lessons home. JAMA Netw Open. 2023;6(9):e2335125. 10.1001/jamanetworkopen.2023.35125 37733341

[14] Khorram-Manesh A Burkle FM Phattharapornjaroen P , et al. The development of Swedish military healthcare system: part II-re-evaluating the military and civilian healthcare systems in crises through a dialogue and study among practitioners. Mil Med. 2021;186(3-4):e442-e450. 10.1093/milmed/usaa364 PMC7665683 33135765

[15] von Elm E Altman DG Egger M Pocock SJ Gøtzsche PC Vandenbroucke JP. Strengthening the reporting of observational studies in epidemiology (STROBE) statement: guidelines for reporting observational studies. BMJ. 2007;335(7624):806-808. 10.1136/bmj.39335.541782.AD 17947786 PMC2034723

[16] Qualtrics. Sample size calculator. 2023. https://www.qualtrics.com/blog/calculating-sample-size/

[17] Waardegedreven zorg: een noodzakelijke basis in de opleiding van zorgprofessionals. 2021. https://www.linnean.nl/inspiratie/bibliotheek/HandlerDownloadFiles.ashx?idnv=1761611

[18] Westerink HJ Steinmann G Koomans M van der Kemp MH van der Nat PB. Value-based healthcare implementation in the Netherlands: a quantitative analysis of multidisciplinary team performance. BMC Health Serv Res. 2024;24(1):224. 10.1186/s12913-024-10712-x 38383368 PMC10882801

[19] Brown T Kātz B , eds. Change by Design : How Design Thinking Transforms Organizations and Inspires Innovation. 1st ed. Harper Business; 2009.

[20] OECD. Measuring Regulatory Performance: A Practitioner’s Guide to Perception Surveys. OECD Publishing; 2012.

[21] Varvasovszky Z Brugha R. A stakeholder analysis. Health Policy Plan. 2000;15(3):338-345. 10.1093/heapol/15.3.338 11012410

[22] Watson CE Leyva-Moral JM Granel N Garro M Navarrete-Reyes L Gómez-Ibáñez R. Understanding value-based healthcare: qualitative insights from nurses and nursing managers in hospital settings. J Nurs Manag. 2025. 2025:9365941. 10.1155/jonm/9365941 PMC12575028 41180586

[23] Wolf A Erichsen Andersson A Wikström E Bååthe F. Untangling the perception of value in value-based healthcare - an interview study. Leadersh Health Serv. 2024;37(5):130-141. 10.1108/LHS-07-2023-0051 PMC11348837 38635293

[24] Huizinga E Idenburg FJ van Dongen TTCF Hoencamp R. Repatriation for diseases or non-battle injuries (DNBI): long-term impact on quality of life. BMJ Mil Health. 2020;166(E):e13-e16. 10.1136/jramc-2019-001194 31005884

[25] Galvin JW Thompson JC Thompson AM , et al. A guide to understanding reimbursement and value-based care in the military health system. Mil Med. 2019;184(3-4):e205-e210. 10.1093/milmed/usy206 30169687

[26] Shelton GHH Ondra S Levin P. Reforming the military health system. Center for a New American Security. 2015. Future of the all-volunteer force series.

[27] Eijsink JFH Fabian AM Vervoort JPM Al Khayat MNMT Boersma C Postma MJ . Value-based health care in western countries: a scoping review on the implementation of patient-reported-outcomes sets for hospital-based interventions. Expert Rev Pharmacoecon Outcomes Res. 2023;23(1):1-13. 10.1080/14737167.2023.2136168 36300427

[28] Makdisse M Ramos P Malheiro D , et al. Value-based healthcare in Latin America: a survey of 70 healthcare provider organisations from Argentina, Brazil, Chile, Colombia and Mexico. BMJ Open. 2022;12(6):e058198. 10.1136/bmjopen-2021-058198 PMC9171220 35667729

[29] Miller T Reihlen M. Assessing the impact of patient-involvement healthcare strategies on patients, providers, and the healthcare system: a systematic review. Patient Educ Couns. 2023;110:107652. 10.1016/j.pec.2023.107652 36804578

[30] Teresi JA Yu X Stewart AL Hays RD. Guidelines for designing and evaluating feasibility pilot studies. Med Care. 2022;60(1):95-103. 10.1097/MLR.0000000000001664 34812790 PMC8849521

[31] Katz G Eit H. Implementing Value-Based Health Care in Europe: Handbook for Pioneers. EIT Health; 2020.

[32] Ndayishimiye C Tambor M Behmane D , et al. Factors influencing health care providers payment reforms in central and eastern European countries. Inquiry. 2024;61:469580241287626. 10.1177/00469580241287626 PMC11526301 39344025

[33] Prusaczyk A Żuk P Guzek M Szafraniec-Buryło S Oberska J. Shifting towards value based healthcare–analysis of theoretical concepts and implementation possibilities in Poland. Journal of Health Policy & Outcomes Research. 2021;(1):16-23.

