# Supplementary material for: Perceived Applicability of Value-Based Healthcare in Military Health Systems: Results From a Pilot Survey Study
Source: Inquiry. 2026 Mar 8;63:00469580261427434. doi: 10.1177/00469580261427434 (PMC12968401; doi:10.1177/00469580261427434)
Supplement: sj-pdf-1-inq-10.1177_00469580261427434 – Supplemental material for Perceived Applicability of Value-Based Healthcare in Military Health Systems: Results From a Pilot Survey Study [file sj-pdf-1-inq-10.1177_00469580261427434.pdf]

Supplementary material 1 (S1) - Overview of Porter's Value Agenda & Linnean Value-Based Healthcare components

1. **Porter's Value Agenda** - the strategic agenda for the change towards high-value healthcare delivery

<table>
<tr><td rowspan="4">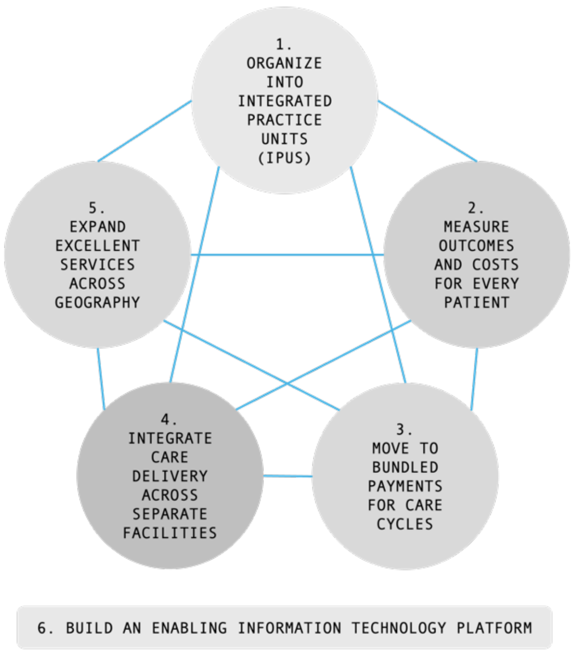
</td><td>Organise care into Integrated Practice Units (IPUs)
IPUs bring together multidisciplinary teams that organise care around a patient's specific condition rather than medical specialties. This structure supports coordinated care across the full cycle, improving outcomes and efficiency.</td></tr>
<tr><td>Measure outcomes and costs for every patient
Systematic measurement of both clinical outcomes and patient-reported outcomes enables transparency and learning. Tracking true costs across the entire care cycle allows organisations to identify where value can be improved.</td></tr>
<tr><td>Move to bundled payments for care cycles
Bundled payments cover all services related to a patient's condition over a defined episode, reducing fragmentation and misaligned incentives. This model rewards outcomes rather than volume, encouraging coordinated, high-value care.</td></tr>
<tr><td>Integrate care delivery across separate facilities
Integration ensures that care is delivered in the most appropriate setting—whether hospital, outpatient clinic, or primary care—without duplications or gaps. It promotes consistent standards and seamless transitions across the system.</td></tr>
<tr><td>Expand excellent services across geographic regions
High-performing units are scaled across locations to provide patients consistent access to high-value care. This expansion leverages expertise and standardised pathways, reducing unwarranted variation.</td><td>Build an enabling information technology platform
A modern IT platform supports integrated care by enabling real-time data sharing, outcome tracking, and coordination across providers. It ensures that all professionals involved in the care cycle have access to the same accurate, patient-centred information.</td></tr>
</table>

2. **Linnean VBHC components** – expanding Porter's Value Agenda

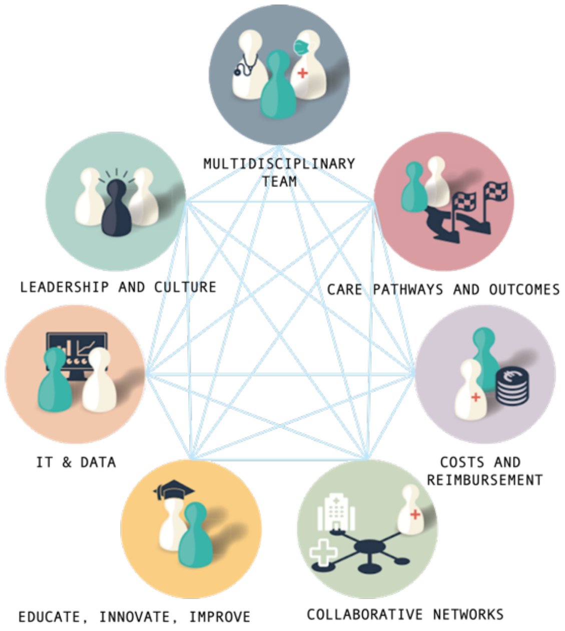

MULTIDISCIPLINARY TEAM
Physicians, nurses, allied health professionals, support staff, managers and **patients** are organised into a **multidisciplinary team.** That team has **joint responsibility** for the **health outcomes** as well as the associated **costs** for the chosen **patient group.** The team is led by a **leader** (or leadership team) that ensures the team receives sufficient resources and is supported. The Board, management and, just as importantly, the staff on the shop floor are also enthusiastically involved!

CARE PATHWAYS AND OUTCOMES
Together, the **patient** and the **healthcare professional** decide which diagnostic and treatment options align best with the **medical necessity** and the **patient's needs**, and are most likely to achieve the desired outcomes in the process of **shared-decision making**. For this purpose, the **standardized outcome set** has been prepared for the specific patient group and visualised in **dashboards**. This is based on the **care pathway,** which has been fully described. This way, the patient and the healthcare professional can see at a glance all the activities that may take place, and when. And because the treatment plan has been drawn up together, it is also immediately clear what diagnostic procedures and treatment are needed.

COSTS AND REIMBURSEMENT
The team knows exactly what the care for the patient group **costs** throughout the entire care pathway. Because it also has insight into the outcomes, the team can make **informed decisions**. It is possible to make comparisons between different diagnostic and treatment options, **prioritise** improvement initiatives, monitor their **impact**, and clarify differences between healthcare professionals and institutions. There are also **Value-Based reimbursement arrangements** or **Value-Based payments** between the healthcare organisation and the health insurer, which stimulate the provision of the best care.

COLLABORATIVE NETWORKS
The team not only works together, but is also actively engaged in **collaborative networks**. Together, they ensure that the patient receives a **total package of care** that is personally tailored to them and streamlined. For the patient, it is clear who the primary caregiver is, who the contact person is, who the scheduler is, and who to go to for 24/7 assistance.

EDUCATE, INNOVATE, IMPROVE
Healthcare professionals who get even better at their jobs every day. Team members understand the idea of Value-Based Health care and do not miss an opportunity to **learn and improve**, from the individual patient, from patient groups, from each other and from others. The results are shared **transparently** with the outside world so that the patient, referrer and colleagues have immediate insight into where the best care is provided for a specific patient (or patient group).

IT & DATA
IT and data systems that help. The relevant data are **findable, accessible, interoperable** and **reusable**. Above all, the data are **reliable, available** in time and **high in quality**. This makes it possible to interpret the data quickly and easily, for example in clear **dashboards**. And, of course, all of this is done in a **secure IT environment**. Accordingly, the data stimulate quality of care, communication with patients and fellow healthcare professionals, as well as innovation and scientific research.

LEADERSHIP & CULTURE
A culture in which the focus is put back on the patient and the best care delivery for that patient. Care delivery has become a **team effort** because team members **share responsibility** for the entire care pathway. To do so, people **collaborate** effectively, are aware of each other's activities, **trust** each other, are **transparent** and open, and **learn from mistakes** (their own or those of others). **The leadership** has **guts** and **ambition**, take **responsibility** and ensure an atmosphere of trust and **safety, learning and improvement.**
