# Supplementary material for: Perceived Applicability of Value-Based Healthcare in Military Health Systems: Results From a Pilot Survey Study
Source: Inquiry. 2026 Mar 8;63:00469580261427434. doi: 10.1177/00469580261427434 (PMC12968401; doi:10.1177/00469580261427434)
Supplement: sj-pdf-2-inq-10.1177_00469580261427434 – Supplemental material for Perceived Applicability of Value-Based Healthcare in Military Health Systems: Results From a Pilot Survey Study [file sj-pdf-2-inq-10.1177_00469580261427434.pdf]

**Supplementary material 2 (S2) - Overview questionnaire VIVIMED Military Medicine conference – workshop**

| ***Variable*** | ***Question*** | ***Answers*** |
|---|---|---|
| RoleMHC | What is your role within military healthcare? | Other / military patient ((potential) care recipient) |
| | | Military healthcare professional (healthcare provider) |
| | | Military leadership (healthcare support) |
| HowManyYearsExp | How many years of experience do you have within Defence? | 0 - 15 years |
| | | 5+ years |
| WhichCountryPre | Which country do you represent? | Czech Republic |
| | | Slovakia |
| | | Hungary |
| | | Poland |
| | | Others |
| ScaleExtFamVBHC | On a scale of 1 (not at all) to 10 (completely), to what extent are you familiar with the concept of value-based healthcare? | 1 |
| | | 2 |
| | | 3 |
| | | 4 |
| | | 5 |
| | | 6 |
| | | 7 |
| | | 8 |
| | | 9 |
| | | 10 |
| FamApplImplemVBHC_CompCountry | Are you familiar with application or implementation of VBHC components in your home country? | Yes |
| | | No |
| PatBeInvolvedOwnPatJourney | Should the patient be involved in his/her own (military) patient journey? | Yes |
| | | No |
| WhereAppVBHC_Total | Where in military healthcare could VBHC be applicable? | Operational Care |
| | | Regular Care |
| | | Not Applicable |
| | Which components of VBHC could be applicable in military healthcare? *Multiple answers can be selected.* | |
| CompAppMHS_MultDiscTeam | multidisciplinary team | Yes |
| | | No |
| CompAppMHS_CarePathOutc | care pathways & outcomes | Yes |
| | | No |
| CompAppMHS_CostsReimb | costs & reimbursements | Yes |
| | | No |
| CompAppMHS_CollNetw | collaborative networks | Yes |
| | | No |
| CompAppMHS_EdInnoImpr | educate, innovate & improve | Yes |
| | | No |
| CompAppMHS_ItData | IT & data | Yes |
| | | No |
| CompAppMHS_LeadCult | leadership & culture | Yes |
| | | No |
| CompAppMHS_NA | N/A | Yes |
| | | No |

| Variable | Question | Answers |
|---|---|---|
| WhatExtDesirVBHC_PartMHS | On a scale of 1 (not at all) to 10 (completely), to what extent do you find it desirable to make VBHC part of your own Military Health System? | 1 |
| | | 2 |
| | | 3 |
| | | 4 |
| | | 5 |
| | | 6 |
| | | 7 |
| | | 8 |
| | | 9 |
| | | 10 |
| WhoImplemVBHC_MHS | Suppose VBHC were to be implemented in our Military Health System. Who do you think should take this initiative? | Direct care team (bottom-up) |
| | | Indirect care team (top-down) |
| | | Both care teams in collaboration with each other |
