# Supplementary material for: Perceived Applicability of Value-Based Healthcare in Military Health Systems: Results From a Pilot Survey Study
Source: Inquiry. 2026 Mar 8;63:00469580261427434. doi: 10.1177/00469580261427434 (PMC12968401; doi:10.1177/00469580261427434)
Supplement: sj-pdf-3-inq-10.1177_00469580261427434 – Supplemental material for Perceived Applicability of Value-Based Healthcare in Military Health Systems: Results From a Pilot Survey Study [file sj-pdf-3-inq-10.1177_00469580261427434.pdf]

Supplementary material 3 (S3) - Outline interactive workshop, with topics & survey questions

Outline workshop	Topic / Question	Items addressed during the interactive workshop
Opening & Introduction	T	Introduction aim of workshop and survey
	T	Introduction of the research team
Background participants to identify healthcare background and military experience	Q	What is your role within military healthcare?
	Q	How many years of experience do you have within defence?
	Q	Which country do you represent?
Mapping familiarity with VBHC concept	T	Visualize Porter's definition of value in healthcare
	Q	On a scale of 1–10, how familiar are you with the concept of VBHC?
	T	Introduction of Value-Based Healthcare (VBHC)
	T	Explanation of VBHC based on Value: person-centred care, outcomes that matter to patients, sustainability; Outcome: maximizing survivability and quality of life in military and civilian care; and Costs: reducing 'waste': efficiency and effectiveness improvements
	T	Transition toward value-based healthcare delivery systems (Porter to Dutch example)
Mapping awareness of application of VBHC in own country	Q	Are you familiar with application or implementation of VBHC components in your home country?
	T	Military health systems versus VBHC: a comparison overview, including 'Patient outcome not equal to patient-reported outcome'
Exploratory question on patient involvement in his/her own (military) patient journey	Q	Should the patient be involved in his/her own (military) patient journey?
Where to apply VBHC and which components to apply	T	Where is VBHC in the Military Healthcare?
	T	How and where to create value for the Military Health (eco-)System?

	Q	Where in military healthcare could VBHC be applicable?
	Q	Which VBHC components could be applicable? (multiple answers)
Level of desirability to make VBHC part of their own MHS, and who should take initiative	T	Introduce framework Design Thinking, explaining the elements: Desirability, Feasibility, and Viability
	Q	To what extent do you find it desirable to make VBHC part of your Military Health System?
	Q	If VBHC were implemented, who should take the initiative?
Closing	T	Closing remarks
	T	Contact information of research team
T = Exploratory topic; Q = Survey question		
