# Supplementary material for: Perceived Applicability of Value-Based Healthcare in Military Health Systems: Results From a Pilot Survey Study
Source: Inquiry. 2026 Mar 8;63:00469580261427434. doi: 10.1177/00469580261427434 (PMC12968401; doi:10.1177/00469580261427434)
Supplement: sj-pdf-4-inq-10.1177_00469580261427434 – Supplemental material for Perceived Applicability of Value-Based Healthcare in Military Health Systems: Results From a Pilot Survey Study [file sj-pdf-4-inq-10.1177_00469580261427434.pdf]

Supplementary material 4 (S4) - Grouping of applicable VBHC components

This table presents a detailed tabulation of how respondents grouped applicable VBHC components, offering insights into the priorities and distribution of these elements across various implementation settings. Each row of the table corresponds to a specific VBHC component, while columns delineate the number and percentage of respondents selecting between one and seven components. This dual perspective is achieved by considering both the overall popularity of each component and the distribution of that popularity across varying levels of engagement with the VBHC framework.

Respondents by applicable group of components	N	%	N	%	N	%	N	%	N	%	N	%	N	%	Total respondents N	Total respondents %	
	1	2,9%	1	2,9%	8	22,9%	4	11,4%	10	28,6%	7	20,0%	4	11,4%	35	100%	
Grouping of applicable components per respondent	1 of 7		2 of 7		3 of 7		4 of 7		5 of 7		6 of 7		7 of 7		Total per single component		Total percentage of all respondents by single component
Single component per respondent	N	%	N	%	N	%	N	%	N	%	N	%	N	%	N	%	
Multidisciplinary team	1	3,3%	0	0,0%	5	16,7%	4	13,3%	10	33,3%	6	20,0%	4	13,3%	30	100,0%	85,7%
Care pathways & outcomes	0	0,0%	1	3,7%	4	14,8%	2	7,4%	10	37,0%	6	22,2%	4	14,8%	27	100,0%	77,1%
Costs & reimbursements	0	0,0%	0	0,0%	3	18,8%	2	12,5%	4	25,0%	3	18,8%	4	25,0%	16	100,0%	45,7%
Collaborative networks	0	0,0%	0	0,0%	0	0,0%	2	11,8%	4	23,5%	7	41,2%	4	23,5%	17	100,0%	48,6%
Educate, innovate & improve	0	0,0%	1	3,4%	6	20,7%	3	10,3%	8	27,6%	7	24,1%	4	13,8%	29	100,0%	82,9%
IT & data	0	0,0%	0	0,0%	3	12,5%	0	0,0%	10	41,7%	7	29,2%	4	16,7%	24	100,0%	68,6%
Leadership & culture	0	0,0%	0	0,0%	3	15,0%	3	15,0%	4	20,0%	6	30,0%	4	20,0%	20	100,0%	57,1%
